# Mobile based surveillance platform for detecting Zika virus among Spanish Delegates attending the Rio de Janeiro Olympic Games

**DOI:** 10.1371/journal.pone.0201943

**Published:** 2018-08-22

**Authors:** Natalia Rodriguez-Valero, Miguel Luengo Oroz, Daniel Cuadrado Sanchez, Alexander Vladimirov, Marina Espriu, Isabel Vera, Sergi Sanz, Jose Luis Gonzalez Moreno, Jose Muñoz, Maria Jesus Ledesma Carbayo

**Affiliations:** 1 ISGlobal, Barcelona Centre for International Health Research (CRESIB), Hospital Clínic (Department of International Health)-Universitat de Barcelona, Barcelona, Spain; 2 Biomedical Image Technology, Electronic Engineering, Universidad Politécnica de Madrid & CIBER-BBN, Madrid, Spain; The University of Hong Kong, CHINA

## Abstract

**Background:**

Zika virus has created a major epidemic in Central and South America, especially in Brazil, during 2015–16. The infection is strongly associated with fetal malformations, mainly microcephaly, and neurological symptoms in adults. During the preparation of the Rio de Janeiro Olympic Games in 2016, members of Olympic Delegations worldwide expressed their concern about the health consequences of being infected with Zika virus. A major risk highlighted by the scientific community was the impact on the spreading of the virus into new territories immediately after the Games.

**Objectives:**

To detect real-time incidence of symptoms compatible with arboviral diseases and other tropical imported diseases among the Spanish Olympic Delegation (SOD) attending the Rio Olympic Games in 2016.

**Methods:**

We developed a surveillance platform based on a mobile application installed in participant’s smartphones that monitored the health status of the SOD through a daily interactive check of the user health status including geo-localization data. The results were evaluated by a study physician on-call through a web-based platform monitoring system. Participants presenting severe symptoms or those compatible with Zika infection prompted an alarm in the system triggering specialized medical assistance and allowing early detection and control of the introduction of arboviral diseases in Spain.

**Summary of the results:**

The system was downloaded by 189 participants and used by 143 of them (76%). Median age was 38 years (IQR 16), and 134 (71%) were male. Mean duration of travel was 19 days (+/-9SD). During the Games the highest accumulated incidence observed was for headache: 6.06% cough: 5.30% and conjunctivitis: 3.03%. The incidence rate of cough during the Olympic Games was 1.1% per day per person, followed by headache 0.8% and 0.4% conjunctivitis or diarrhea. In our cohort we observed that non-athletes experienced more incidence of symptoms, except for incidence of cough which was the same in the two groups (1.1%). No participants reported symptoms fulfilling Zika definition case.

**Conclusion:**

Our system did not find cases fulfilling Zika definition amongst participants of the SOD during the Games, consistent with limited cases of Zika in Rio during the Games. The app showed good usability and the web based monitoring platform allowed to manage infectious cases in real-time. The overall system has proven to serve as a real-time surveillance platform for detecting symptoms that could be present in tropical imported diseases, especially arboviral diseases, contributing to the preparedness for the introduction of vector borne-diseases in non-endemic countries.

## Introduction

Zika disease is a flaviviral infection transmitted mainly by *Aedes spp*. mosquitoes of tropical and subtropical areas, but other vectors can be involved in the transmission such as Culex quinquefasciatus[1]. The incubation period, after being bitten by an infected mosquito, ranges from 2 to 15 days[2]. The symptoms are similar to other arboviral infections as dengue or chikungunya and the currently accepted clinical definition case is: patient presenting with a rash, with or without fever and at least one of the following signs and symptoms: arthralgia or myalgia or non-purulent conjunctivitis/hyperemia[3] lasting up to one week, being considered the major symptoms to recognize Zika infection[2]. Nevertheless, it is believed that the majority of the cases are asymptomatic, up to 60% reported in some studies made in Micronesia[4,5].

From 2007 to 2014 there have been several outbreaks in different Pacific Islands. During those outbreaks the link between Zika and Guillain-Barre was already described[6], highlighting the avidity of Zika virus for neurological cells.

Moreover, since 2015 a big continental outbreak is taking place in Central, South America and Caribbean countries. WHO declared Zika as a public health emergency mainly because an increasing number of fetal malformations observed during Zika outbreak. Later, in 2016, Zika virus was strongly associated with fetal malformations[7,8]. The fact that Zika can be sexually transmitted increases the odds of spreading[8], and this is also a differential feature compared to other arbovirus.

To date, other territories, including some Asian countries, have started to report more Zika and microcephaly cases[9,10,11].

Brazil has been one of the most stricken countries by Zika. There have been twenty-times more cases of microcephaly and increasing mortality amongst newborns compared with the previous years since the Zika epidemic began[12]. Rio de Janeiro is one of the territories that had registered the highest number of Zika infections until Summer of 2016 [13].

Since the 2016 Olympic Games were planned in Rio de Janeiro during the Zika epidemic, a big concern was raised in terms of individual health consequences and spreading of the virus to other susceptible territories. Moreover, an open-letter wrote by more than 100 academics was sent to World Health Organization suggesting cancelling the Games [14]. Nevertheless, predictive models claimed that the possibility of being infected and consequently spreading the virus was very low and it was decided that the Games would continue as planned[15,16,17].

Zika outbreak during Olympic Games gave us the opportunity to do digital participatory surveillance (DPS) in order to perform outbreak discovery, determine incidence of infectious diseases during the Games and also search for infectious disease predictors[18] through a system similar to other initiatives as Influenzanet for seasonal epidemics of Influenza [19] or Healthy Cup App during the FIFA world Cup 2014[20].

The aim of this project was to detect real-time daily incidence of symptoms compatible with Zika virus acute syndrome and other arboviral diseases among the SOD attending the Rio Olympic Games in 2016 through an integrated surveillance platform, to ensure proper and early treatment and avoid spreading of infectious diseases during and after the Olympic Games 2016.

## Materials and methods

The SOD was formed by athletes and other professionals working in the Olympic Games under the umbrella of the Spanish Olympic Committee.

During the summer of 2016, our research group invited all members of the Spanish Delegation to download a Smartphone software application called “OlymTRIP” designed ad-hoc for the event and gave them written instructions about how to use it. The app also provided information and advice about Zika infection. In a daily basis the app asked for the health state at the personally set time. In case of feeling unwell the app provided the contact of the doctors of the Spanish Olympic Committee who in addition could track in real-time the health status of the SOD members using a web-based platform. Prior to use the app, a disclaimer was accepted by all participants. The app can be publicly downloaded for Android on Google Play. If a researcher wants to use it and replicate the experiment a code will be given to unblock it after signing a non disclosure agreement. In addition, the matrix can be also accessed if requested.

**Fig 1:**
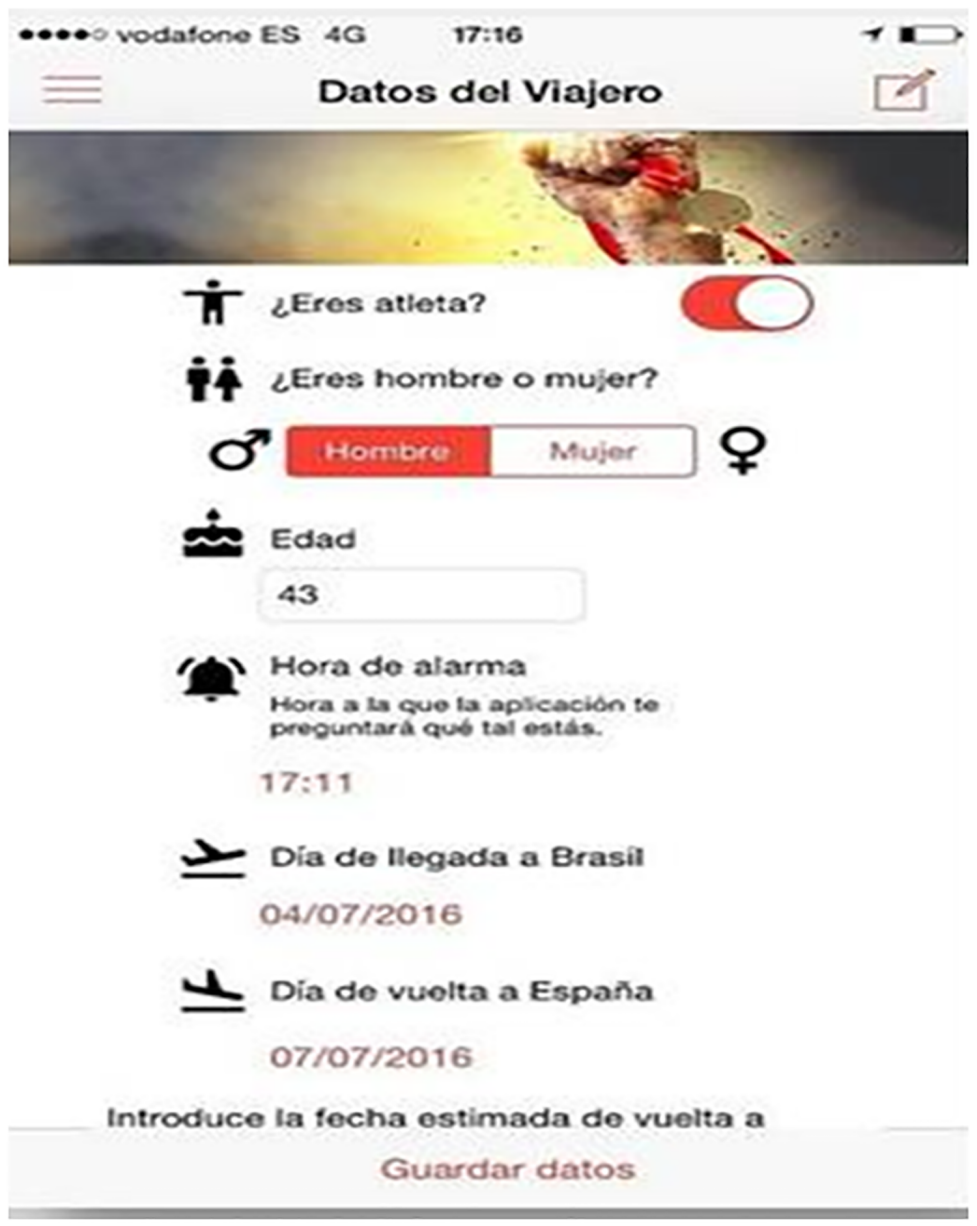
OlympTRIP home screen and traveler data.

**Fig 2:**
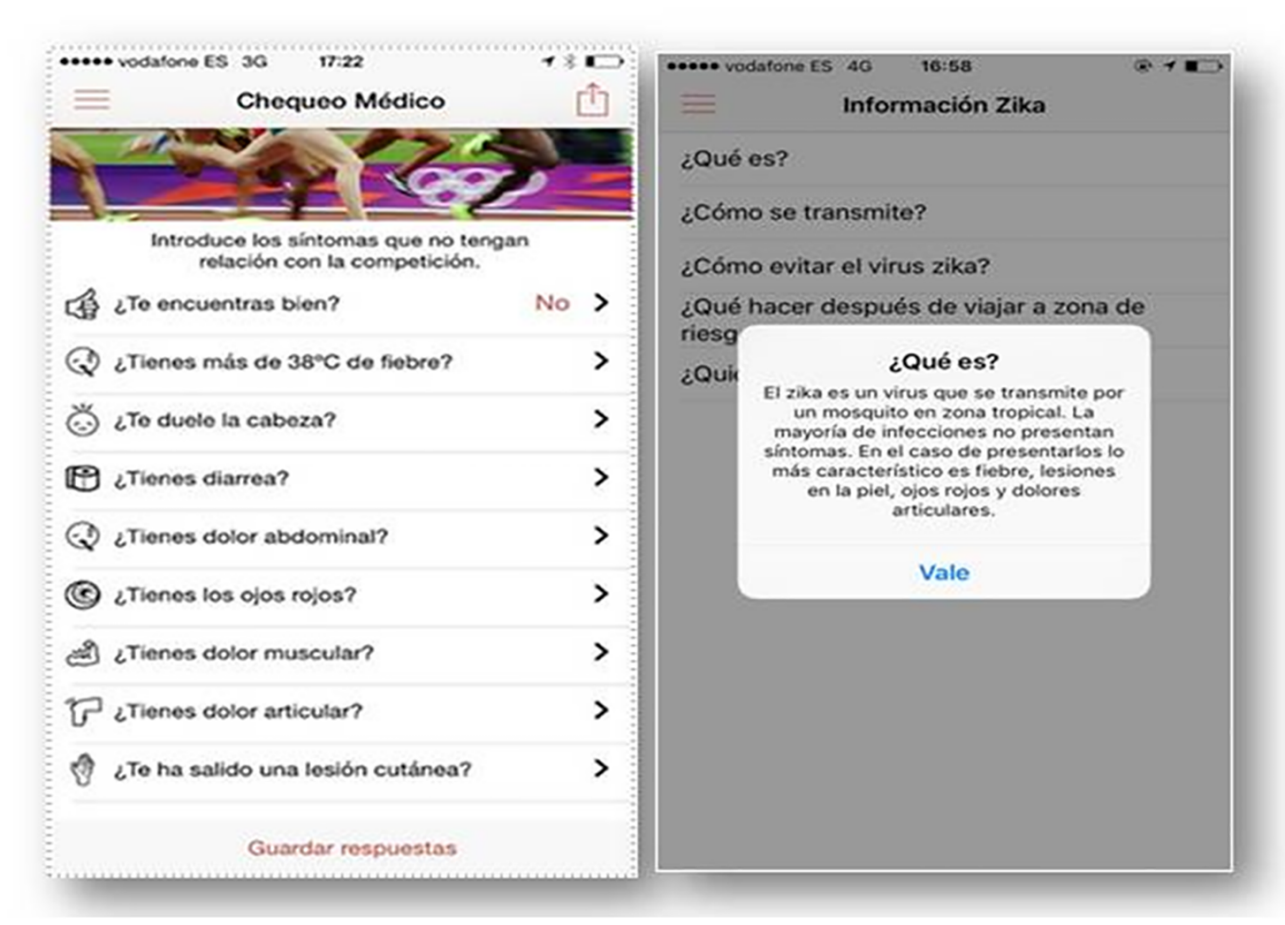
OlympTRIP daily check status and Zika information.

OlympTRIP was able to register anonymously basic characteristics of each participant: age, sex, sport discipline, athlete status, and arrival and departure dates from Brazil “Fig 1”. Every day the app interacted with the user at a predetermined time asking about their health status and in case of athletes the app asked for competition days, recording the answer and the date of every interaction. In case of not feeling well the app would request about different symptoms recording the presence of cough, diarrhea, abdominal pain, arthralgia, headache, cutaneous lesions, conjunctival hyperemia, fever, and also an approximate GPS location of each check and optionally a picture associated with the symptoms in case of cutaneous lesions “Fig 2”. The symptoms were selected to detect the most common tropical imported diseases to ensure their detection.

**Fig 3:**
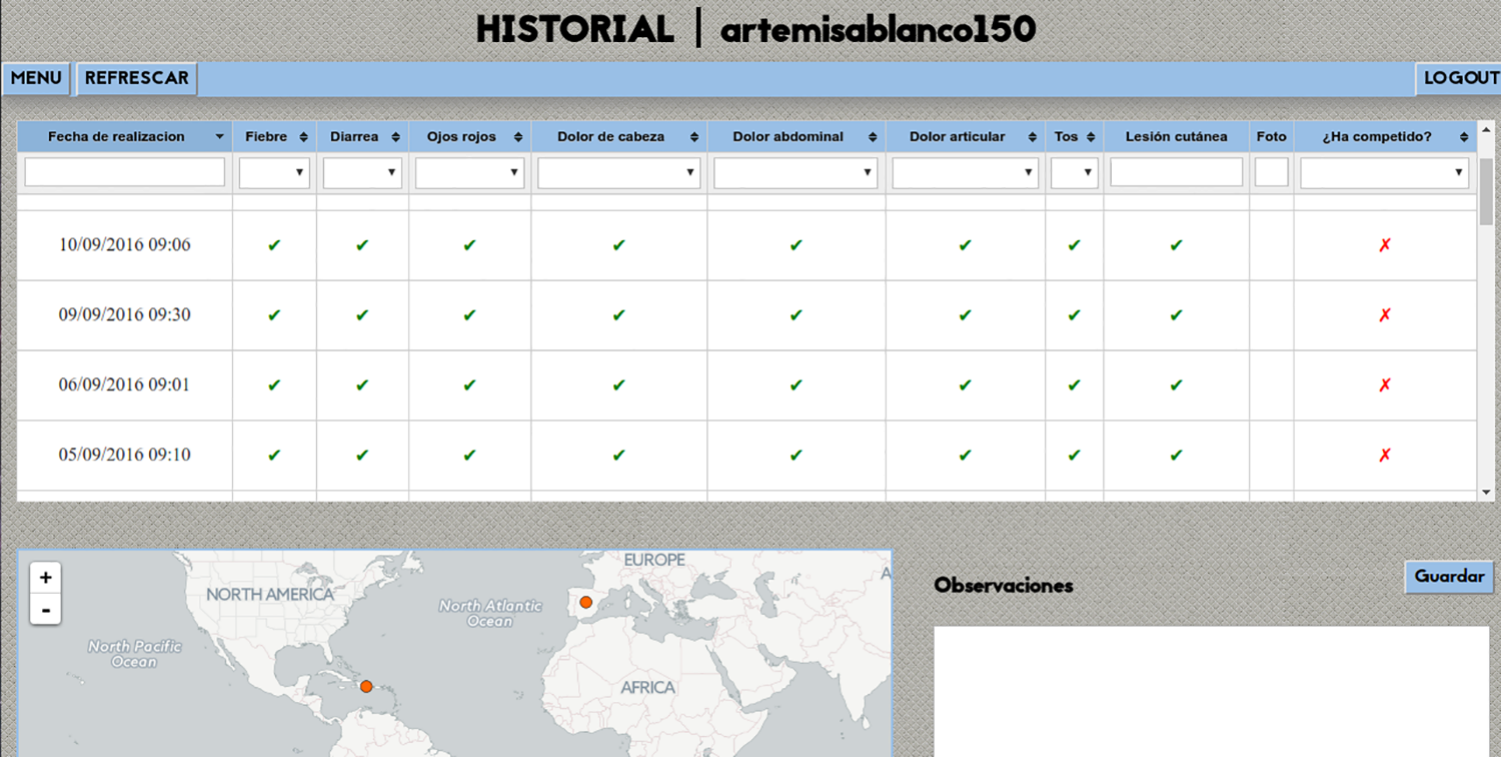
Web-based surveillance platform.

All the information was stored in a data base in the cloud, processed in real-time in a back end system “Fig 3”, and showed in a dashboard in a dedicated website to be monitored and evaluated by our study physician and the medical team of the Spanish Olympic Committee in order to control the health status of the participants as well as detecting one or more cases with similar characteristics. The system ensured privacy by design as no private information was stored that could enable match the entries in the database with the individual participants. The medical team was the only personnel that could decipher the systems codes into individuals in order to contact them and ensure the proper health status. The system automatically identified symptoms of arboviral diseases, and possible clusters of cases or outbreaks.

The Data was analysed with Stata 14.2 (Stata Corporation, College Station, TX, USA). Differences between proportions were compared using the Pearson´s *chi-squared* test or Fisher’s exact test depending on type of variables. For continuous variables, Student’s T-tests were used to compare the groups. To calculate incidence of symptoms, accumulated incidence or rate incidence was used, defined as number of cases in a given period adding all periods of observation. The incidence rate has been expressed in persons-day units. We consider a result as statistical significance when we have obtained a p-value below 0.05. Secondary objectives were to estimate the potential risk of spreading of Zika in Spain after the Games taking into account the number of participants that fulfill Zika definition and potentially carrying the disease. Participants presenting symptoms compatible with arboviral diseases would be identified and contacted upon return to establish measures to control the introduction of the disease.

All research has been approved by the authors’ Institutional Review Board (IRB) "Comité de Ética de la Investigación con Medicamentos" of Hospital Clinic Barcelona (reference HCB/2016/0503) and the clinical investigation has been conducted according to the principles expressed in the Declaration of Helsinki. A written disclosure was sent to the participants along with the App and it had to be accepted in order to start using the App.

Data is available upon request because contain potentially identifying patient/athlete information. The request should be made to the Spanish Olympic Committee (correo@coe.es) and the dataset will be shared after permission of the Spanish Olympic Committee. The Spanish Olympic Committe granted the field permission in order to conduct the investigation.

## Results

Of all members of the Spanish Olympic Delegation 189 participants downloaded the app. Of them, 71% were male, and median age was 38 years (IQR 16). Among the users 29% were athletes and 71% other professionals associated with the Olympic Delegation (managers, journalists, coaches, physiotherapists etc.). The median of days of the stay in Brazil of the cohort were 19 (IQR = 9) and mean days of competition was not possible to calculate due to the missing data of answers in the daily questionnaire. Athletes median stay was 17 days (IQR = 11) and no differences with other participants were found (p = 0.70). Results displayed in “Table 1”.

**Table 1:** Demographic and travel-related characteristics of the study participants.

Baseline	N = 189
**Sex**	
Male	71% (134/189)
Female	29% (55/189)
**Age (years)**	
Median (IQR)	38 (16)
Mean (SD)	40 (12)
**Athletes/Non-athletes**	29% (54/189)/ 71% (135/189)
**Lenght of stay (days)**	
Median (IQR)	19 (9)
Mean (SD)	18 (11)

In terms of usability, 142 (75%) of participants whom downloaded the app used it. 46 of them (25%) did not use the app not even once. Among the participants who used the app, 68 (48%) made 10 or more checks of symptoms at different days (daily checks), and 74 (52%) made less than 10 daily checks.

Three participants started to use the app when they experienced symptoms. Of all participants that downloaded the app, they used it a mean of 8.79 days (SD = 7). Athletes used the app significantly fewer days than other users (6.4 ±6.01 days vs. 9.79±7.17 days; p 0.012). But no differences in the mean of days not using the app were seen between athletes and non-athletes. “Table 2”

**Table 2:** Usability of the app among participants.

Usability	N = 189	p-value^ψ^
**App use**	142 (75%)	
**Daily checks**		
**No checks**	46 (24%)	
**Less than 10 daily checks**	74 (39%)	
**More than 10 daily checks**	68 (36%)	
**Days of app use**^a^		
**All participants**	8.79 (6.99)	
**Non athletes**	9.79 (7.17)	0.01
**Athletes**	6.47 (6.01)	
**Days of no use of the app**^a^		
**All participants**	11.44 (8.95)	
**Non athletes**	10.79 (9.54)	0.20
**Athletes**	12.93 (7.28)	

^**ψ**^ comparison between Athletes and non athletes.

^a^ Mean (SD).

**Fig 4:**
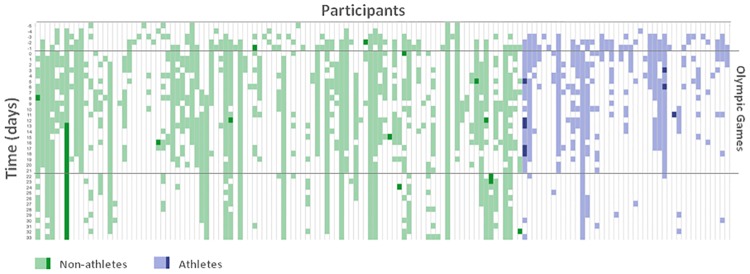
Daily checks by participant during the period of observation. Each column corresponds to one participant, each row correspond to one day. Green colors correspond to non-athlete participants, and blue to athlete participants. Light colors correspond to daily checks when participants were not presenting any symptom and dark colors when participants were feeling any of the described symptoms.

The symptoms reported by the participants were distributed in dates -1 to +11 with respect to the Olympic Games dates “Fig 4”.

Concerning the GPS information, 40% of the recordings from all the interactions with the app were valid. Of the total of users that had at least one interaction with the App (148), 61.5% had at least one valid GPS recording through their travel.

The most incident symptoms among participants, calculated for all days using the app, were cough 7.38%, headache 6.04%, conjunctivitis 2.68%, and diarrhea 2.68%. During the Games the accumulated incidence of headache was of 6.06%, cough 5.30%, conjunctivitis 3.03% and the accumulated incidence of traveler’s diarrhea was 2.27%. The rest of accumulated incidences during the Games period were: abdominal pain 0.76%, joint pain 1.52%, cutaneous lesions 0.76%, and fever 0.76%. A 5% (8/149) of the users had one symptom during their stay and the percentage of participants having 2 or more symptoms was 7% (10/149). No significant differences in percentages of symptoms were found stratifying by sex or athlete status.

The highest calculated incidences rates during the whole period of study were: 2.2% person-day for cough and 0.7% for headache. In our cohort we observed that non athletes had significantly more incidence of symptoms: 1% more incidence per day in terms of cough and 0.2% more incidence of headache “Table 3”.

**Table 3:** Incidence rates of symptoms during all the period of study by athlete status.

Symptom	Incidence Rate Global Incidence Rate^a^	Athlete Incidence Rate	Non-athlete Incidence Rate	Rate Difference*	95% CI
**Diarrhea**	0.4%	0	0.5%	0.5%	-
**Abdominal pain**	0.2%	0	0.2%	0.2%	-
**Joint pain**	0.3%	0	0.3%	0.3%	-
**Headache**	0.7%	0.6%	0.8%	0.2%	(0.6–1.1)
**Cutaneous lesions**	0.2%	0	0.3%	0.3%	-
**Conjunctivitis**	0.4%	0	0.5%	0.5%	-
**Cough**	2.2%	1.4%	2.4%	1.0%	(0.3–2.5)
**Fever**	0.3%	0	0.4%	0.4%	-

*rate difference between athlete incidence and non-athlete incidence.

^a^cases person/per day,CI:confidence interval

The incidence rate of cough during the Olympic Games was 1.1% per day per person, followed by headache 0.8% and 0.4% conjunctivitis and diarrhea. In our cohort we observed that non-athletes experienced significantly more incidence of symptoms than athletes: 0.1% more cough and also 0.5% more incidence per day of headache than athletes “Table 4”.

**Table 4:** Incidence rates of symptoms during the Games and stratified by athlete status.

Symptom	Incidence Rate Global Incidence Rate^a^	Athlete Incidence Rate	No athlete Incidence Rate	Risk difference
**Diarrhea**	0.4%	0	0.5%	0.5%
**Abdominal pain**	0.08%	0	0.1%	0.1%
**Joint pain**	0.2%	0	0.3%	0.3%
**Headache**	0.8%	0.4%	0.9%	0.5%
**Cutaneous lesions**	0.2%	0	0.3%	0.3%
**Conjunctivitis**	0.4%	0	0.5%	0.5%
**Cough**	1.1%	1.1%	1.1%	0.1%
**Fever**	0.2%	0	0.3%	0.3%

^a^cases person/day, RR:rate ratio

## Discussion

A total of 76% of all the participants who downloaded the app used it; this reflects that the app was friendly and easy to use. The good engagement, compare to other initiatives as Health Cup App[20], could be due to: concern about Zika among SOD members, the survey length that was short, daily push notifications and also the possibility to answer offline, Moreover, three participants used the app just in the day when they presented symptoms, adding value to the app, increasing its sensitivity to detect patients among participants, seen also in other DPS[21]. Athletes used the app less than other users, probably because of the amount of stress and responsibilities during the Games compared to other participants. A simple installation protocol was defined and documented to enable that the SOD could install the app appropriately. However, due to the complex logistical operation that represents the Olympic Games with SOD participants traveling in different dates and to different places, it is possible that some did not receive their personal credentials to download the app.

There were no differences in terms of downloading the app being an outdoor or indoor participant. Some disciplines with few members were more inclined to download the app, perhaps because of the small size of the group allowing communication between them. It is also very possible that the athletes simply did not download the app or report their health status as much as non-athletes due to the personal context- the Games were probably among the most important moments of their life, where concentration and stress is maximum. In terms of symptoms cough was the most reported symptom (11 patients), probably caused by winter infections as influenza concordant to other mass gathering events in winter in Brazil such as FIFA world cup where cough and respiratory syndromes where the most incident[20]; followed by headache in 9 cases, which isolated could be a symptom related to conditions other than infections as jet-lag or stress. The above figures are consistent with the incidence rate, or the risk of having a symptom during the Games, being the incidence of cough the highest rate, 1.1% per day per person, with no differences between athletes and non-athletes. The rest of symptoms were more incident in the non-athlete group, this could be because the non-athletes used the app more than athletes, and also because they reported more symptoms, we have to take into account that the stay for non-athletes was more prolonged (2 days more without significant difference) and the conditions on stress and concentration of the athletes could have influence. Through our study we cannot evaluate if the difference is due to the different behavior between the two groups and whether this could influence the difference in observed incidence rates compared to Influenzanet which was able to detect differences in behavior adding questions about the daily life of the participants[19]. Adding questions to our system could be done because of its flexibility in order to determine predictors better in future events.

Not a single patient fulfilled Zika definition case (patient presenting with a rash, with or without fever and at least one of the following signs and symptoms: arthralgia or myalgia or non-purulent conjunctivitis/hyperemia)[3], and this is probably because there were limited cases in Rio during the Games. Absence of cases in general was probably due to the low presence of mosquitoes and mosquito bites, the inappropriate weather for mosquito presence during August in Rio de Janeiro and the efforts of Brazilian authorities before the Games to control the vector; which is reflected in the number of cases in Rio population during July and August 2016, 14154 Zika cases were recorded in Rio province during July and 0 Zika cases between 32 and 37th week of 2016 corresponding to August[22,23,24]. Additionally a serological study of a sample of the SOD was made resulting in no cases of Zika detected among the members of the SOD tested after the Olympic Games[25] checking the proper accuracy and sensitivity of our system detecting Zika[21].

The app allowed our group and the medical team in Rio, to detect suspect cases and outbreaks real-time, also by localization and by symptoms over time. It also allowed to the SOD doctors displaced to Rio, to monitor the health status of all the participants who downloaded the app and reaching the patients as soon as symptoms appeared in order to avoid complications of common and tropical and infectious diseases.

As other DPS[21] our system has biases such as bias of selection that could be improved by allowing multiusers in the app and thus trying to have more age representation (children and elderly). Even though there is lack of microbiological validation of the symptoms reported to the App we correlated our findings with traditional epidemiological tools checking our system accuracy and sensitivity. Finally, we should add more questions to the survey, other than medical, in order to search better infectious disease predictors and adjust for confounders.

## Conclusions

Our system did not find cases fulfilling Zika definition amongst participants of the SOD during the Games, as there were limited cases of Zika in Rio during the Games. With more than 76% of the SOD that downloaded the app using it, we believe this type of system composed by an app and a back end system with an appropriate monitoring website platform is suitable for massive surveillance of health symptoms, since it is easy for medical doctors to monitor the status of many patients in parallel and in real time.

The overall system has proven to serve as a real-time surveillance platform for detecting symptoms of tropical imported tropical diseases, especially arboviral diseases, contributing to the preparedness for the introduction of vector borne-diseases in non-endemic countries. The system is also scalable and flexible and it is a tool of awareness about tropical infectious diseases among the public.

## References

[1] Guedes DRD, Paiva MHS, Donato MMA, Barbosa PP, Krokovsky L, Rocha SWS, et al Zika virus replication in the mosquito Culex quinquefasciatus in Brazil. Emerg Microbes Infect. 2017;6(8). 10.1038/emi.2017.59 PMC5583667 28790458

[2] Musso D, Gubler DJ. Zika Virus. Nature. 2016;11(1):10–20.

[3] European Centre for Disease Control. Case definition Zika virus. 2016;21 July.

[4] Musso D, Nhan T, Robin E, Roche C, Bierlaire D, Zisou K, et al Potential for Zika virus transmission through blood transfusion demonstrated during an outbreak in French Polynesia, November 2013 to February 2014. Eurosurveillance. 2014;(4 2014):14–6. 10.2807/1560-7917.es2014.19.14.20761 24739982

[5] Duffy MR, Chen T-H, Hancock WT, Powers AM, Kool JL, Lanciotti RS, et al Zika virus outbreak on Yap Island, Federated States of Micronesia. N Engl J Med. 2009;360(24):2536–43. 10.1056/NEJMoa0805715 19516034

[6] Cao-Lormeau V, Blake A, Mons S, Lastere S, Roche C, Vanhomwegen J, et al Guillain-Barré Syndrome outbreak caused by ZIKA virus infection in French Polynesia. Lancet. 2016;6736(16):In press. 10.1016/S0140-6736(16)00562-6 PMC5444521 26948433

[7] De Brito CAA, Cordeiro MT. One year after the Zika virus outbreak in Brazil: From hypotheses to evidence. Rev Soc Bras Med Trop. 2016;49(5):537–43. 10.1590/0037-8682-0328-2016 27812646

[8] Blázquez A-B, Saiz J-C. Neurological manifestations of Zika virus infection. World J Virol. 2016;5(4):135–43. 10.5501/wjv.v5.i4.135 27878100 PMC5105046

[9] European Centre for Disease Control. Rapid Risk Assessment. Zika virus disease epidemic. Ninth update, 28 October 2016. Stockholm. ECDC; 2016.

[10] Schuler-Faccini L, Ribeiro EM, Feitosa IML, Horovitz DDG, Cavalcanti DP, Pessoa A, et al Possible Association Between Zika Virus Infection and Microcephaly—Brazil, 2015. MMWR Morb Mortal Wkly Rep. 2016;65(3):59–62. 10.15585/mmwr.mm6503e2 26820244

[11] Mlakar J, Korva M, Tul N, Popović M, Poljšak-Prijatelj M, Mraz J, et al Zika Virus Associated with Microcephaly. N Engl J Med. 2016;160210140106006. 10.1056/NEJMoa1600651 26862926

[12] França GVA, Schuler-Faccini L, Oliveira WK, Henriques CMP, Carmo EH, Pedi VD, et al Congenital Zika virus syndrome in Brazil: a case series of the first 1501 livebirths with complete investigation. Lancet. 2016;388(10047):891–7. 10.1016/S0140-6736(16)30902-3 27372398

[13] Pan American Health Organization/World Health. Zika-Epidemiological Report Brazil. Washington D.C. PAHO;

[14] Open Letter to Dr. Margaret Chan, Director-General, WHO (Copied to the International Olympic Committee) The Washington Post. 13 May 2016. https://www.washingtonpost.com/news/to-your-health/wp-content/uploads/sites/26/2016/05.

[15] Codeço C, Villela D, Gomes MF, Bastos L, Cruz O, Struchiner C, et al Zika is not a reason for missing the olympic games in rio de janeiro: Response to the open letter of dr attaran and colleagues to Dr Margaret Chan, director—General, WHO, on the zika threat to the olympic and paralympic games. Mem Inst Oswaldo Cruz. 2016;111:414–5. 10.1590/0074-02760160003 27304097 PMC4909043

[16] Massad E, Tan SH, Khan K, Wilder-Smith A. Estimated Zika virus importations to Europe by travellers from Brazil. Glob Health Action. 2016;9(1):31669. 27193266 10.3402/gha.v9.31669 PMC4871896

[17] Grills A, Morrison S, Nelson B, Miniota J, Watts A, Cetron MS. Projected Zika Virus Importation and Subsequent Ongoing Transmission after Travel to the 2016 Olympic and Paralympic Games—Country-Specific Assessment, July 2016. Morb Mortal Wkly Rep. 2016;65(28):711–5. 10.15585/mmwr.mm6528e1 27442184

[18] Pagliari C, Vijaykumar S. Digital Participatory Surveillance and the Zika Crisis: Opportunities and Caveats. PLoS Negl Trop Dis. 2016;10(6):1–5. 10.1371/journal.pntd.0004795 PMC4905668 27294787

[19] Guerrisi C, Turbelin C, Blanchon T, Hanslik T, Bonmarin I, Levy-Bruhl D, et al Participatory syndromic surveillance of influenza in Europe. J Infect Dis. 2016;214(January):S386–92. 28830105 10.1093/infdis/jiw280

[20] Leal Neto O, Dimech GS, Libel M, de Souza WV, Cesse E, Smolinski M, et al Saúde na Copa: The World’s First Application of Participatory Surveillance for a Mass Gathering at FIFA World Cup 2014, Brazil. JMIR Public Heal Surveill [Internet]. 2017;3(2):e26 Available from: http://publichealth.jmir.org/2017/2/e26/10.2196/publichealth.7313 PMC5438444 28473308

[21] Wójcik OP, Brownstein JS, Chunara R, Johansson MA. Public health for the people: Participatory infectious disease surveillance in the digital age. Emerg Themes Epidemiol. 2014;11(1):1–7. 24991229 10.1186/1742-7622-11-7 PMC4078360

[22] Secretaria de Vigilància em Saúde. Boletim Epidemiológico. Vol. 47–no31. Brasilia; 2016.

[23] Secretaria de Vigilància em Saúde. Boletim Epidemiológico. Vol. 47–no34. Brasilia; 2016.

[24] Secretaria de Vigilància em Saúde. Boletim Epidemiológico. Vol. 47–no33. Brasilia; 2016.

[25] Rodriguez-Valero N, Borobia AM, Lago M, Sánchez-Seco MP, De Ory F, Vázquez A, et al Zika virus screening among Spanish team members after 2016 Rio de Janeiro, Brazil, olympic games. Emerg Infect Dis. 2017;23(8):1426–8. 10.3201/eid2308.170415 28628450 PMC5547782

